# Exploring the digital psychology of environmental sustainability: the mediating influence of technological innovation in advanced physical education development in China”

**DOI:** 10.1186/s40359-024-01673-1

**Published:** 2024-03-27

**Authors:** Wenhao Liu, Ruilin Xu, Songpu Li

**Affiliations:** 1 School of Physical Education (Main Campus), Shangqiu Normal University, 476000 Shangqiu, China; 2 School of Physical Education (Main campus), Zhengzhou University, 450001 Zhengzhou, China

**Keywords:** Advanced physical education, Technological innovation, Environmental sustainability, Structural equation modeling, China

## Abstract

The study aims to investigate the precise processes by which the advancement of physical education and technological progress leads to ecological conservation efforts within China’s distinctive socio-cultural and economic framework. Acknowledging the pivotal role that economic advancement plays in a nation’s environmental sustainability, this research utilizes cross-sectional quantitative data gathered using a five-point Likert scale survey. The sample size included 503 undergraduate students from Zhengzhou, China, and structural equation modeling was utilized to analyze the data. The study investigates how technology progress influences the relationship between compatibility, environmental sustainability, and the relative benefits of physical education. It fills the gap in the literature by illuminating how technical innovation and advanced physical education development contribute to China’s pursuit of a sustainable environment. The findings emphasize the critical significance of higher physical education in fostering environmental sustainability. Furthermore, the research indicates that students participating in more rigorous physical education programs tend to possess a more well-rounded and mature mindset. This mindset is essential for healthy and long-lasting mental development, motivating individuals to critically consider environmental sustainability. The study provides valuable theoretical and practical insights that can be applied to enhance environmental sustainability in the country.

## Introduction

Advanced physical education goes beyond essential fitness and sports teaching. Health education, fitness promotion, and sports skill acquisition are among its many events and educational experiences that promote holistic development [1]. Advanced physical education programs often include nutrition, mental health, and environmental sustainability to give learners an integrated view of the value of active lifestyles in individual and societal health. However, higher physical education programs at colleges and universities focus on more profound research in physical education, sports biology, and related fields [2]. These programs may offer specialized coursework, research opportunities, and practical experiences to prepare learners for careers in coaching sports, physical fitness management, and rehabilitation, as well as promote physical education scholarship and creative thinking [3].

In the current era, countries are experiencing an increase in environmental issues such as pollution, deforestation, and climate change due to the effects of globalism, which is unsuitable for people worldwide [4]. Remarkably, countries with higher populations suffer from the consequences of globalization as people face environmental problems that limit their productivity and potential [5]. Given that, some global institutions and forums are working to eradicate this problem of environmental crisis worldwide [6]. However, as a collective approach, these institutions have not been successful in implicating the containment of globalism until now.

Similarly, being a large country by population, China is suffering from environmental issues that are not good for the health of her population [6]. However, it is critical to recognize that the Chinese are deeply concerned about their industrial and production growth and development, which have also increased their environmental anxieties. According to various surveys, the environmental problems in China seem to be uncontrollable [7]. On the positive side, the Government of China is taking remarkable initiatives to contain environmental problems, which are severe hurdles in the way towards the environmental suitability of China [8–9] as this instability has detrimental consequences not only for the people in China but also, it is harmful to the populations residing in the neighboring countries of China [10].

Previous studies demonstrate that the stability of the environment simultaneously brings progress in the community and business sectors. At the same time, the instability is considered an influencing factor for disturbing the lives of human beings in the countries. It is the duty of the government in such circumstances to offer substitutes for insufficient leadership and the ramifications to support development objectives for raising public awareness of environmental sustainability [11]. It is therefore believed that if the people are not provided with the awareness related to environmental sustainability, it would be ineffective for the government to regulate the policy for a sustainable environment as well as provide a better environment to the people [12–13]. Previous studies have not adequately explored the vital role of sustainable technology innovation in enhancing environmental sustainability for the benefit of society. Sustainable technology innovation refers to technological advancements prioritizing environmental preservation while improving productivity and people’s well-being in a country [14].

Undoubtedly, technology plays a critical role in shaping the world’s dynamics, and supporting globalism has been identified as a fundamental cause of environmental issues [15]. Some earlier studies perceived globalism as a problem responsible for environmental degradation and societal harm [16]. Consequently, these studies focused on management practices related to achieving competitive advantages in environmental sustainability through sustainable development goals. This study is crucial to understanding how China is accomplishing environmental sustainability while improving physical education and technology. China, a worldwide economic giant, presents tremendous environmental concerns, requiring a better knowledge of economic growth, technological advancement, and sustainable practices. Although economic growth is essential for environmental sustainability, more research is needed on the impact of advanced physical education development and technology innovation. This study addresses this gap using a cross-sectional quantitative technique and a five-point Likert scale survey. The paper acknowledges the importance of structural equation modeling in analyzing the complex linkages between compatibility, environmental sustainability, technological advancement, and physical education advantages.

The present study explores the relationship between physical education, sustainable technological innovation, and environmental sustainability. To the researchers’ knowledge, no previous studies have specifically examined environmental sustainability in China. Therefore, this study seeks to understand how compatibility, simplicity, and the mediating role of sustainable technological innovation influence environmental sustainability in China. Additionally, the study acknowledges the significant role of physical education in China’s environmental sustainability. It is therefore anticipated that this study would close the research gap in the literature and expand it by offering new findings from a previously unexplored context. For sustainable development, different institutes work for different reasons to protect the environment and develop sustainability [17]. The expected contribution of this study will be best suited for the management and all its stakeholders to improve the environment to provide a reliable alternative to protect the environment from destruction.

### Research questions

These research questions must be answered. First, how does technological innovation affect Chinese advanced physical education development and environmental sustainability? This study examines how technology advances help or impede the incorporation of sustainable practices into school curricula and everyday routines in China to understand how environmental awareness develops. Second, how does compatibility, as impacted by technological advancement, affect the perceived environmental sustainability advantages of advanced physical education? This inquiry examines how compatibility—the connection between technology advancements and advanced physical education program goals—affects students’ perceptions of their advantages. This study examines how technology compatibility improves advanced physical education’s ability to implant environmental values and behaviors to optimize sustainable development education tactics. Finally, considering technology involvement, how can demanding physical education programs affect students’ environmental awareness and sustainability attitudes? This study examines the relationship between physical activity, technology use, and environmental consciousness to determine how advanced physical education programs help Chinese youth develop environmentally responsible behaviors, informing sustainability-focused educational policies.

Addressing a literature gap, this study investigates variables crucial for China’s environmental sustainability, particularly in a dynamic and changing global landscape. Environmental issues in China are escalating, leading to the outbreak of various diseases and causing widespread societal damage [8]. The severity of these environmental problems calls for immediate attention, as the future of China’s environment will face even greater challenges. Environmental sustainability is paramount for the growth of industries and communities [18–19].

## Literature review

The literature review is divided into three main sub-sections that focus on the key themes of the study related to the advancement in physical education linked with technological innovation and environmental sustainability. Additional discussions are presented in the mentioned sub-sections.

### Relationship of relative advantage, technological innovation, and environmental sustainability

The environmental sustainability crisis has become a global concern, adversely affecting human life and society [20]. International institutions are crucial in promoting globalization for environmental sustainability [21]. Despite this, challenges in advancing environmental sustainability are attributed to these institutions’ negligence of stakeholder responsibility [22–23]. Global institutions must create policies and initiatives to ensure a positive environmental impact [24] Apart from governments, other factors significantly contribute to shaping the environmental landscape and achieving sustainability [25–26]. The responsibility of management is integral in ensuring societal parameters adhere to environmental standards. However, in some underdeveloped countries, management struggles to deliver environmentally friendly operations [27–28]. The intersection of modern technology and innovation offers promise for improving environmental performance [17, 23]. Failing to meet management responsibilities and societal ethics poses a challenge to achieving environmental sustainability [11]. In North America, businesses and society are trying to advance environmental sustainability [29]. Organizational culture influences technology use and its outcomes [19]. Observing ethical standards is essential for embracing business practices focused on environmental sustainability. The path to environmental sustainability requires strategic planning guided by ethical standards [11].

### Relationship of compatibility, technological innovation sustainability, and environmental sustainability

Compatibility is pivotal in advancing technology toward sustainability [5, 30]. It significantly impacts societies, elevating living standards by fostering sustainable technological development that meets people’s needs [23]. However, achieving and maintaining compatibility is challenging and demands substantial effort [31–32]. Competence is essential for technological innovation aligned with sustainable development. Stakeholders are responsible for integrating and implementing compatibility measurements for technological growth [5]. In developed countries, environmental sustainability is a core focus for organizations operating under corporate social responsibility [32–33]. They endeavor to integrate competence within organizations to drive technological innovation for sustainable development [34–35]. For example, in countries like the United States and Japan, technological advancements are continually introduced and adopted to enrich society [36–37]. However, it’s crucial to ensure that such technological progress aligns with environmental sustainability to avoid long-term societal damage [38]. Stakeholders must adhere to ethical guidelines in technology innovation to foster compatibility that’s not detrimental to society in the long run [39–40].

### Relationship of simplicity, physical education, technological innovation sustainability, and environmental sustainability

The eternal nature of technology must be user-friendly and understandable, highlighting the significance of simplicity in technology innovation [41]. Nonetheless, several traditional organizations aimed primarily at revenue generation, overlooking the ethical responsibility to the environment [42–43]. Ethical guidelines are crucial to address complications in technology use and drive environmental sustainability [44–45]. Companies not adhering to environmental sustainability guidelines might struggle to sustain themselves in the market and society [46]. Global drivers are pivotal in providing simple, sustainable technology development guidelines for societal understanding and environmental preservation [15]. Technology innovation and corporate social responsibility’s alignment are fundamental to ensuring technology development remains reliable without affecting the environment [47–48].

While customers in Canada value sustainability in products, organizations in certain global regions may neglect corporate social responsibility guidelines, hindering sustainable development [23, 49]. In remote areas in India, sustainable development goals are not implemented, posing a challenge to achieving corporate social responsibility standards [11]. Collaboration between society and organizations in understanding corporate social responsibility guidelines is pivotal for a more sustainable environment [50–51].

Furthermore, effective management should support sustainable technology innovation while deterring practices that go against sustainable development goals [52]. Physical education is key for individual physical and mental development, fostering a motivated and skilled workforce [11, 53]. Proper understanding and adoption of sustainable development goals and physical education significantly contribute to environmental sustainability.

### Mediating role of technological innovation sustainability

Technology innovation sustainability focuses on utilizing innovative technology to address societal concerns and ensure the benefit of society [14]. It plays a vital role in the sustainability of the environment and its development, serving as one of the key factors for sustainability [23]. Organizations must responsibly use technological innovation in line with corporate social responsibility to benefit society [11, 54]. Compatibility, an essential factor for societal benefit, helps individuals gain critical skills necessary for organizational sustainability [55]. Management’s competence in implementing sustainable development goals aids in organizational and societal advancement [56–57]. In the global context, organizations worldwide should innovate technology for sustainability and benefit from its attractiveness and productivity [30]. Japan’s dedication to sustainable technology illustrates how it contributes to environmental sustainability.

### Theoretical framework

**Fig. 1:**
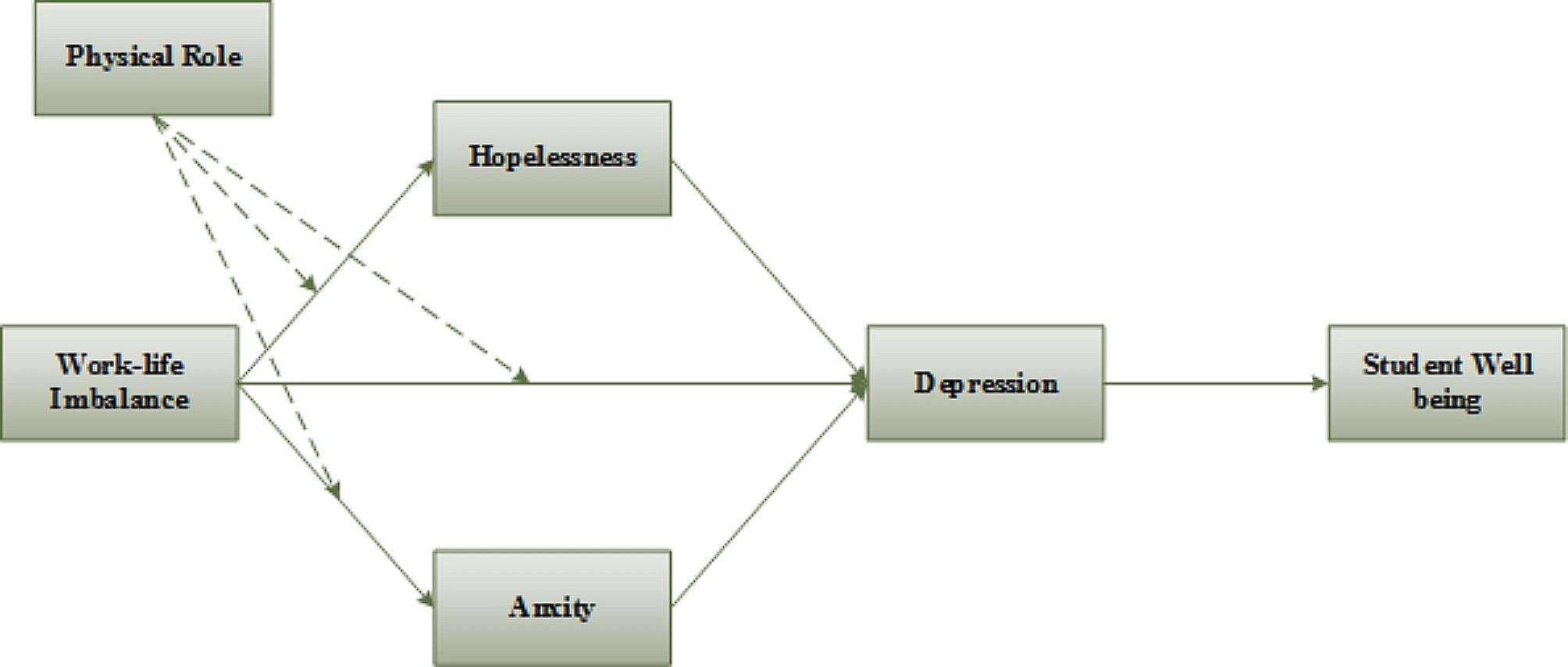
Research framework. *Source*: Author’s work

The research framework of this study is grounded in the ‘Environment Sustainability Theory.’ The environmental theory highlights that it is society’s responsibility to protect environmental progress by not compromising the values of the coming generation [58]. This theory was presented with a deep concern about the environmental problems that demonstrate that sustainability must be provided to the environment because, otherwise, it damages the whole atmosphere of society. In this way, this theory highlights that factors including efficient working, sustainable environment, and responsible use of resources are critical for the sustainability of the environment [59]. Concerning all variables influencing the sustainability of the environment, this study takes the newly identified variables such as physical education, compatibility, and simplicity as important for the sustainability of the environment. In addition, the framework of this study also considers technological innovation sustainability as a mediator in the relationship between relative advantage and compatibility simplicity to environmental sustainability (Fig. 1). Importantly, no earlier study was conducted to test the relationship of these variables to determine the role of higher physical education development and technological innovation toward a sustainable environment in China. Therefore, the theoretical framework of this study provides a new approach to the literature and practices by considering the significant variables for the greater contribution in the light of environment sustainability theory.

### Research hypotheses

The following research hypotheses have been explored within the given research framework, categorizing them into different research categories, i.e.,

- Synergies Among Technology, Physical Education, and Environmental Sustainability.

*H1. There is a positive relationship between perceived relative advantage of advanced physical education development and technological innovation.*

*H2. There is a positive relationship between perceived relative advantage and environmental sustainability.*

*H3. There is a positive relationship between technological innovation sustainability and environmental sustainability.*

- Compatibility in Advanced Physical Education, Technology, and Environmental Sustainability.

*H4. There is a positive relationship between perceived compatibility of advanced physical education development and technological innovation sustainability.*

*H5. There is appositive relationship between perceived compatibility of advanced physical education development and environmental sustainability.*

- Simplicity’s Influence on Technological Innovation and Environmental Sustainability.

*H6. There is a positive relationship between perceived simplicity of advanced physical education development and technological innovation sustainability.*

*H7. There is a positive relationship between perceived simplicity of advanced physical education development and environmental sustainability.*

*H8. There is a positive relationship between physical education and environmental sustainability.*

- Technological Innovation Sustainability Mediating Pathways.

*H9: Technological innovation sustainability mediates the relationship between relative advantage and environmental sustainability.*

*H10: Technological innovation sustainability mediates between the relationship of compatibility and environmental sustainability.*

*H11: Technological innovation and sustainability mediate the relationship between simplicity and environmental sustainability.*

Preliminary studies have examined how technology innovation and enhanced physical education synergistically support China’s environmental sustainability [60–61]. Technology and physical education have been shown to affect environmental awareness and behavior, but a few research has examined their combined influence in China [62–63]. Earlier research demonstrates that technical advances may solve environmental problems, but without a corresponding emphasis on environmental education, they may worsen them [64–65]. This research attempts to overcome this gap by showing how physical education and technology might improve environmental sustainability in China. This study helps politicians, educators, and stakeholders create successful environmental awareness and behavior campaigns.

## Methodology

To attain the study’s objectives, a quantitative approach was employed, and a questionnaire was devised following a comprehensive literature review. The questionnaire was structured into two parts. The first part gathered respondents’ demographic data. The second segment utilized a Likert scale (five-point) to evaluate variable relationships based on hypotheses. In this study, four scale items focused on relative advantage and compatibility were based on Sin et al. [66] and Farrell & Salomer [67] methods, respectively. Meanwhile, another four scale items denoted as physical education was adopted from Farrell & Saloner [67] approach. Lastly, the remaining three scale items, which represent simplicity, technological innovation sustainability, and environmental sustainability, were adopted from their methods, respectively [27, 68–69].

### Data collection process

This study utilized a cross-sectional data collection method, due to its ease and reliability. This method is particularly beneficial as it saves time when implemented through surveys. The target population was sampled, and detailed introductions to the study’s purpose and objectives were provided with the questionnaires. The questionnaires were distributed upon obtaining respondents’ consent, stressing the need for accurate, anonymous responses. The researcher’s email address was provided to improve the respondents’ understanding of the questionnaire. From a total of 675 questionnaires sent out, 536 questionnaires were collected, and only 503 questionnaires were analyzed because 33were rejected due to inaccuracies. The sample size consisted of 503 undergraduate students from Zhengzhou, China. The researchers used smart PLS to conduct the SME of the quantitative data.

### Instrumental design and procedure

The study used a variety of well-established instruments to assess psychological and well-being components to grasp the factors fully. A 7-item Beck Anxiety Inventory (BAI) was used to measure anxiety [70]. The 7-item Beck Depression Inventory-II (BDI-II) [71] assessed depression. The 4-item Beck Hopelessness Scale (BHS) [72] measured hopelessness. The physical role has 6 items, whereas Student Well-being used 8 items, designed by Clarke et al. [73] used the Warwick-Edinburgh Mental Well-being Scale (WEMWBS). Finally, work-life Balance with 5 items. Each instrument has high validity and reliability ratings, ranging from 0.80 to 0.90, showing that they assess the desired components. Cronbach’s alpha values for all items are over 0.70, suggesting strong internal consistency and stability of readings. Subscales in the instruments allow for a more fine assessment of particular aspects of each construct, enriching and deepening knowledge of the psychological and well-being dimensions under study.

### Data analysis procedure

For structural equation modeling (SEM) with small to medium-sized samples and complicated models, Smart PLS was chosen as the primary analytical tool [74]. Smart PLS can handle non-normal data distributions, formative measurement models, and robust estimations even with smaller sample sizes due to the study’s interdisciplinary nature and multiple latent variables [75]. Smart PLS allows for the evaluation of direct and indirect effects within the suggested conceptual framework, allowing for a complete analysis of factor connections [76]. This research uses Smart PLS to understand the complicated relationship between these factors and add to sustainability and education literature. Smart PLS also conforms with current SEM methodological advances, guaranteeing robust analysis and interpretation of research data.

The study used SEM, a robust statistical method for analyzing complicated interactions between the studied variables. SEM allows the evaluation of direct and indirect effects, model fit, and reliability using observable and latent variables in a theoretical framework. This work used SEM with Smart PLS software to analyze complicated models with small to medium-sized samples. SEM assessed model fit metrics, including the goodness-of-fit index (GFI) and comparative fit index (CFI), to assess the conceptual framework’s validity.

The foundation of a structural equation model is established by an assessment of the structural model and measurements. The first criteria used to assess measurement models are indicator reliability, discriminant validity, convergent validity, and internal consistency reliability. SEM modelling with partial least squares (PLS-SEM) involves verifying the convergence of latent variables. The idea of precision is fundamental to the validation of measuring equipment. Encapsulating this notion is the word “validity,” which questions the accuracy of measuring goods or structures. To what extent the construct is measuring its intended constructs is the central question. For a statistical analysis to be conducted, it is necessary to demonstrate statistical validity, more especially construct validity. Build validity is aided by content validity and face validity, which are achieved by prior research or expert judgements. Convergent validity is one of two concept validity types. Average variance extracted (AVE) supports the latent construct’s validity by measuring its indicator variation extraction. Convergent validity requires the components to represent the latent notion. AVEs above 0.50 suggest convergent validity. The average of an indicator’s squared loadings divided by the construct’s total indicators yields its AVE. AVE is natural for a construct’s communality. When working with several constructs, discriminant validity, the second aspect of construct validity, becomes very necessary. A construct’s discriminant validity may be defined as the degree to which it differs from other conceptions in terms of empirical evidence.

## Findings

This research illuminates the complex relationship between advanced physical education development, technology innovation, and environmental sustainability in Chinese higher education. Educational institutions are vital in encouraging environmental awareness and sustainable behaviors among students as the urgent need for sustainable practices to meet global environmental concerns grows. This research examined how advances in physical education and technology contribute to environmental sustainability objectives to inform policymakers, educators, and environmental activists. The study helps explain the complex dynamics of environmental sustainability efforts in education by evaluating important factors and finding relevant processes.

The research included 503 undergraduate students from Zhengzhou, China. To ensure academic and demographic diversity, stratified random sampling was used to choose participants. A balanced gender distribution was achieved with 53% male and 47% female participation. The participants were 19–25 years old, with the majority 20–22 age group. The sample also included students from humanities (25%), natural sciences (20%), social sciences (30%), and engineering (25%). Participants have provided informed permission. These demographic features assured a broad sample of academic interests and viewpoints, bolstering the study’s robustness and generalizability.

### Convergent validity

**Fig. 2:**
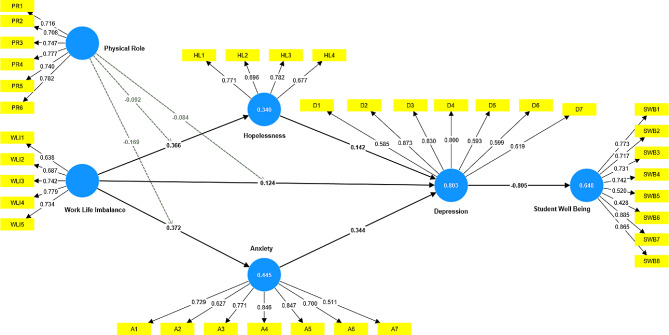
Measurement model. *Source*: Author’s work

This section of the study presents the findings of the convergent validity analysis, which aimed to determine the relationship between the scale items utilised for each variable in this study (Fig. 2). The factor loadings for each scale item were determined, and the results indicate that every scale item has a factor loading beyond the recommended threshold of 0.60, as stated by [77]. Likewise, the composite reliability (CR) was assessed, and the findings indicated that all values exceeded 0.70 (Table 1). In addition, the study determined the average variance extracted (AVE) values. The results reveal that all values exceed the required threshold of 0.50, as indicated by [78]. Indeed, according to these findings, there is clear composite reliability in scale items.

**Table 1:** Factor Loadings, CR, and AVE

Factors	Items	Outer Loading	VIF	Cronbach Alpha	CR	AVE
Anxiety	A1	0.729	1.557	0.848	0.885	0.529
	A2	0.627	1.420			
	A3	0.771	2.593			
	A4	0.846	4.276			
	A5	0.847	4.637			
	A6	0.700	1.461			
	A7	0.511	1.255			
Depression	D1	0.585	1.557	0.830	0.874	0.504
	D2	0.873	3.704			
	D3	0.830	3.557			
	D4	0.800	2.184			
	D5	0.593	1.747			
	D6	0.599	1.676			
	D7	0.619	1.721			
Hopelessness	HL1	0.771	1.580	0.718	0.822	0.537
	HL2	0.696	1.580			
	HL3	0.781	1.746			
	HL4	0.677	1.127			
Physical Role	PR1	0.718	1.282	0.844	0.881	0.554
	PR2	0.705	1.684			
	PR3	0.746	1.795			
	PR4	0.776	1.922			
	PR5	0.740	2.015			
	PR6	0.776	2.190			
Student Well Being	SWB1	0.773	2.404	0.866	0.894	0.523
	SWB2	0.717	4.417			
	SWB3	0.731	4.610			
	SWB4	0.742	2.102			
	SWB5	0.520	2.043			
	SWB6	0.428	1.996			
	SWB7	0.885	4.684			
	SWB8	0.865	4.524			
Work Life Imbalance	WLI1	0.638	1.883	0.765	0.841	0.515
	WLI2	0.687	1.946			
	WLI3	0.743	2.923			
	WLI4	0.779	3.067			
	WLI5	0.734	1.341			

### Discriminant validity

This study presents the discriminant validity results, which were assessed using the HTMT method. The purpose was to establish the differentiation between the scale items for each variable. It was determined that each value of discriminant validity was below 0.90, which is the indicated threshold for clear discriminant validity (Table 2). This study demonstrates distinct discriminant validity between the scale items employed for each variable.

**Table 2:** Discriminant Validity (HTMT)

	A	D	HL	PR	SWB	WLI
Anxiety	**–**					
Depression	0.810					
Hopelessness	0.589	0.723				
Physical Role	0.526	0.861	0.577			
Student Well Being	0.624	0.861	0.506	0.477		
Work Life Imbalance	0.670	0.820	0.689	0.866	0.668	

### Partial least square– structure equation modeling

**Fig. 3:**
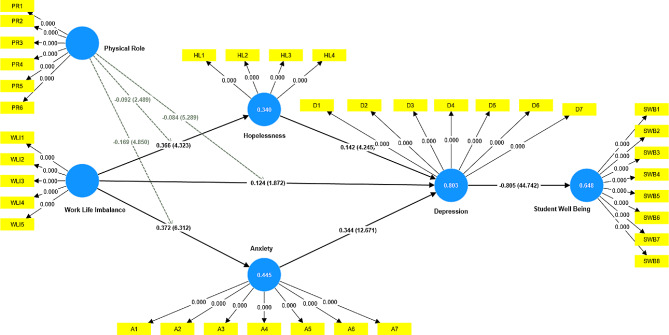
Structural model. *Source*: Author’s work

This section elaborates the relationship of the hypotheses. Initially, H1 was examined to determine its significance. The current findings indicate that RA has a substantial impact on TIS (β = 0.452, t = 6.938, *p* = 0.000), thus confirming the acceptance of H1. The significance of H2 was evaluated, and the results demonstrate that RA is significant on ES (β = 0.294, t = 3.943, *p* = 0.000). Therefore, H2 is considered valid. Moreover, the significance of H3 was assessed and the results indicate that TIS has a substantial impact on ES (β = 0.594, t = 7.545, *p* = 0.000). Therefore, H3 is accepted. Further, the significance of H4 was assessed using a test. The results indicate that CP has significant effects on TIS, with a coefficient (β) of 0.326, a t-value of 4.872, and a p-value of 0.000. Therefore, H4 is accepted. Besides, the significance of H5 was assessed, revealing that CP has a substantial impact on ES (β = 0.130, t = 1.981, *p* = 0.048). Consequently, H5 is accepted as well. Sixthly, regarding the H6, based on the results, SP is significant on TIS (β = 0.124, t = 2.648, *p* = 0.008), and H6 is accepted. Next, H7 was subjected to testing in order to identify its significance. The results indicate that SP has a noteworthy impact on ES, with a β coefficient of 0.112, a t-value of 2.434, and a p-value of 0.012. Therefore, H7 is confirmed to be valid. Additionally, the significance of H8 was assessed through testing. The results prove that PE has a noteworthy impact on ES, with a coefficient of 0.156, a t-value of 2.261, and a p-value of 0.018. Therefore, H8 is confirmed as valid, as illustrated in Fig. 3.

Additionally, the interaction between the mediating hypotheses was examined. The relationship between RA and ES is mediated by TIS, with a β coefficient of 0.268, a t-value of 5.885, and a p-value of 0.000. Consequently, H9 is also accepted. Second, according to the results, TIS mediates the relationship between CP and ES (β = 0.193, t = 3.643, *p* = 0.000). Therefore, H10 is supported. Third, according to the results, TIS mediates the relationship between SP and ES (β = 0.074, t = 2.336, *p* = 0.020). Therefore, H11 is supported.

## Discussion

Climate change, resource depletion, and ecological deterioration have made environmental sustainability a worldwide problem. In response to these issues, education has become more critical in encouraging sustainability and ecologically responsible behavior. Advanced physical education and technical innovation in higher education may foster environmental sustainability. Advanced physical education programs teach pupils about health, well-being, and environmental stewardship, while technology developments help solve environmental problems. This conversation examines the linkages between advanced physical education, technological innovation, and environmental sustainability to understand their effects on sustainable development.

The results of H1 demonstrate a significant relationship between relative advantage and technological innovation sustainability. This hypothesis supports the Innovation Diffusion Theory, which emphasizes perceived advantages in innovation uptake and sustainability. If advanced physical education instructors see considerable benefits in adopting sustainable technology and practices, they are more inclined to include them. The observed association between relative advantage and technological innovation sustainability confirms the theory’s predictions and shows how perceived benefits promote sustainable schooling methods. Technological innovation sustainability essentially helps attain environmental sustainability, in case it is itself attained through the strategies that support it to a greater extent, as suggested by Hosseini et al. [14] in their study. In this way, the stakeholders and the people involved in technological innovation must work together to sustain technology. For example, in Japan, technology is considered an effective tool for sustainability because innovations are more concerned with sustainability-related goals [14, 42]. Therefore, organizational skills must be designed to provide effective and efficient technological innovation that should help sustain the overall environment. However, as technological innovation may also harm the environment, organizations must focus on improving the quality of technology to move further in innovation and its sustainability for the benefit of the environment and society.

The results of H2 demonstrate a significant relationship between relative advantage and environmental sustainability. Theory of Planned Behavior (TPB) states that attitudes, subjective standards, and perceived behavioral control affect behavior intentions. TPB explains the considerable association between relative advantage and environmental sustainability in Hypothesis 2, demonstrating the significance of perceived advantages in altering people’ intentions to embrace sustainable education methods. Sustainability of the environment can be easily maintained if the relative advantage is provided in the target market for the development of the community [11]. Indeed, the organizational culture and the skills of the stakeholders are considered important for the betterment of the environment because the environment depends on them. Moreover, it is emphasized that if society wants to grow productively, it will bring more opportunities if it provides a more productive environment for the people [13]. However, it is not an easy process as it demands a greater understanding between all the stakeholders to attain such higher-level goals.

The results of H3 demonstrate that there is a significant relationship between technological innovation sustainability and environmental sustainability. The Ecological Modernization Theory states that technical breakthroughs may promote cleaner, more efficient technologies and practices, hence improving environmental sustainability. Innovative technologies that improve resource efficiency, waste reduction, and environmental effects support environmental sustainability in advanced physical education. Undoubtedly, the innovation of technology would be more effective for the sustainability of the environment when it is designed to provide sustainable development [43]. Furthermore, the responsibility of organizations is to innovate technology to satisfy the customers’ ineffective ways to provide the maximum benefit. Therefore, in Korea, technology is designed to sustain the environment [16]. The results of H4 and H5 demonstrate a significant relationship between compatibility, technological innovation sustainability, and environmental sustainability. It is important to understand that for sustainable development, the role of society and the organization is important to consider because, without the concept of these both, it would be difficult to achieve sustainability [23].

On the one hand, it is the organization’s responsibility to develop the technology for the satisfaction of the customers to provide maximum benefit to society. However, on the other hand, it is the customers’ responsibility to utilize that technology effectively in a way that would not harm the environment [52]. As a result, a greater benefit would be provided to society, and a maximum outcome would be expected for sustainable development. The cooperation between society and the organization is important because this corporation provides more effective and reliable resources for working sustainably. Significantly, the purpose of an organization is to achieve success in society. In this way, the innovative use of technology and very responsible management would help organizations grow in the target market with the guidelines of sustainable development goals [55, 79].

The results of H6 and H7 highlight a significant relationship among simplicity, technological innovation sustainability, and environmental sustainability. According to Social Cognitive Theory (SCT), personal, contextual, and behavioral variables interact in a dynamic, reciprocal manner to impact behavior. For technological durability and ecological sustainability in modern physical education growth, the apparent simplicity of ethical procedures and technology may affect attitudes and behaviors. Incorporating sustainable innovations into programs is more probable when professionals in the field see them as easy to execute. In this regard, the role of simplicity is critical to understand because it provides the opportunity for the target market to develop effectively for providing the responsible and understandable use of products and services for the sustainability of the environment [23, 51, 80]. Moreover, the technology and the customers contribute a lot to the sustainability of the environment because they are more related to technological innovation as innovation is for their satisfaction. Similarly, if the people of a society are provided with effective technology for their satisfaction and utilization, it would provide more comprehensive and more reliable opportunities for maximum benefit [55, 81]. Indeed, the fair use of technology can lead society in a direction that would benefit sustainable development. Importantly, sustainable development would lead organizations to the advanced level of fairly using the technology as it is being used in Japan. In Japan, society is more reliable and dependent on technology. However, the innovation of organizations is made in a way that would not harm the environment and its sustainability [55].

The results of H8 demonstrate a significant relationship between physical education and environmental sustainability. The Biophilia Hypothesis posits that people are naturally drawn to nature, which affects their health and behavior. Environmental exposure and outdoor activities may help pupils connect with nature in physical education. This relationship may raise environmental awareness and respect for ecological protection. Thus, increasing involvement in physical education programs, especially those that include outdoor activities and environmental education, may improve environmental sustainability attitudes and behaviors. It is critical to determine that sustainable development is needed in society because society is more dependent. However, if people are provided with proper physical education, it would help to develop sustainability and reliability to a greater extent for greater benefit [23, 82]. Importantly, the organizations working for physical education in the public sector provide the opportunity to understand the environment. In this way, the organizations are supposed to perform the critical activities for sustainable development through reliable physical education. The results of H9 highlight the significant mediation of technological innovation sustainability between relative advantage and environmental sustainability.

Similarly, the opportunity must be provided for the relative advantage concerning the innovation of technology to develop a sustainable environment for the benefit of the people. It is critical to understand that unless and until people are provided with the right opportunities in the field of technology, it will not be easy to sustain environmental conditions, as discussed in the study of [83]. Therefore, the fair use of technology is important concerning the relative advantage to achieve maximum benefit [14]. Moreover, sustainability is to develop a more reliable and adaptable atmosphere for better community conditions. According to the study of Dathe et al. [5], the responsibility of the stakeholders is to provide effective management skills for relative advantage to work for a sustainable environment.

The results of H10 highlight the significant mediation of technological innovation sustainability between compatibility and environmental sustainability. The Resource-Based View (RBV) implies that organizations may gain a lasting competitive advantage by using their distinctive resources and skills. This research defines compatibility as how well technology advancements fit into educational settings. Technology is more likely to be embraced and incorporated into educational programs if it fits the educational setting. As technology advances get embedded in organizational infrastructure and procedures, they become more sustainable. Indeed, compatibility is one of the success factors contributing to sustainable development and achieving maximum benefit from it [84]. However, if the technology is developed with proper compatibility, it would be more effective and more reliable for the sustainability of the environment. In Japan and Korea, the organizations innovated the technology under environmental sustainability guidelines, not harming mother nature and providing that affection to the people with the new technology [85]. Therefore, technology must be developed effectively and designed to provide a reliable alternative for sustainable development. The results of H11 highlight the significant mediation of technological innovation sustainability between compatibility and environmental sustainability. According to the Contingency Theory, organizational performance relies on internal capabilities and external environmental conditions. This research defines compatibility as how well technology advancements fit the educational setting and organizational culture. Technological advances are more likely to be embraced and incorporated into educational programs if they fit current practices and procedures. Technological innovation sustainability improves when innovations are integrated into organizational structure and operations. Increasing the sustainability of technical breakthroughs allows educational institutions to implement environmentally sustainable policies and initiatives, improving environmental sustainability results. According to Gorbunova & Hiner [86], it is critical to understand that the factor of simplicity also influences the sustainability of the environment. Indeed, the people with the simplicity approach are not rigid to a greater extent. However, they are demanding sustainability in the environment, as discussed in the study of Le Roux & Nagel [42]. It is noted that in such geographical areas where simplicity is one of the people’s important values, the technology is designed for the maximum satisfaction of the needs of the people [14, 87–88]. Therefore, simplicity must be considered as one of the effective variables for the sustainability of the environment.

## Theoretical and practical implications

### Theoretical implications

The rapidly developing area of educational technology provides cutting-edge instruments and approaches that may significantly improve the efficacy of educational programs meant to advance ecological sustainability. Educators can use digital platforms, modeling, and hands-on instruction to help them develop a deeper understanding of sustainability concepts by incorporating technological resources into developed physical education expansion [89]. Furthermore, physical education supports ecology by referencing theories related to the matter, such as the socio-ecological model and self-determination theory. According to the socio-ecological paradigm, environmental, social, and personal elements all interact to impact an individual’s behavior [90]. Advanced physical education courses that address these multi-level factors and provide a comprehensive comprehension of the relationship between human wellness and the natural world may act as a catalyst for changes in environmental behavior. Furthermore, by highlighting the significance of independence, skill, and connection in fostering intrinsic motivation and behavioral engagement, self-determination theory sheds light on the motivational components of physical education [91]. Through the alignment of sustainability issues objectives with the tenets of self-determination theory, educators may enable students to assume responsibility for their education and cultivate a personal obligation towards environmental stewardship. When combined with the assistance of instructional technology, this innate desire may help students develop a long-lasting commitment to sustainable behaviors.

This study provides significant theoretical implications regarding the role of higher physical education development and technological innovation in attaining a sustainable environment in China. In this regard, it is important to understand that environmental unsustainability is a critical problem in China, leading its population and business sectors to decline. Indeed, it is the government’s responsibility to integrate with all the relevant people to develop strategies in an effective way for the containment of the problems related to the environment. On the one hand, the earlier studies have discussed agriculture tourism’s role in environmental sustainability in China. On the other hand, no study, in particular, addressed the literature gap identified in the current study. In this way, the current study provides significant theoretical implications for solving the environment-related problems in China with the mediating role of environmental sustainability.

Hence, this study demonstrates the relationship between different variables used in its theoretical framework to suggest a complex and rigid relationship between different variables, which are important for the sustainability of the environment in China. However, this relationship is rather explained straightforwardly in the study. It is designed to provide a guideline to the stakeholders to get detailed information and work effectively for the progress of environmental sustainability in China. Hence, it is important to discuss theoretically the appropriate relationship between technological innovation sustainability and environmental sustainability effectively. Respectively, all the stakeholders working for environmental sustainability must work effectively to integrate all the related discussions of the current study in an effective way to reach a better conclusion or solution to this matter. As a result, the technological innovation in China would lead the organizations to carry out a more effective strategy for working in the right direction regarding environmental sustainability.

### Practical implications

This study addresses the empirical gap and provides significant implications regarding the role of higher physical education development and technological innovation toward a sustainable environment in China. In this regard, it is important to understand that the government’s management of an organization and its stakeholders is significant for the sustainability of the environment in modern times. The reason behind it is that each government works according to the guidelines and values of corporate social responsibility (CSR) which may provide a shield to the problems emerging from globalization and its outcomes. In this manner, this study highlights that stakeholders must understand the role of higher physical education in providing effective skills and development in the community to work as the guidelines for corporate social responsibility to improve society. Indeed, organizations are responsible for environmental sustainability. Therefore, the responsibility of the organizations is to work for the relative advantage and competency for improvement in CSR.

Similarly, if the organizational culture supports CSR, it would be more effective and reliable to innovate in terms of technology with the consideration of CSR. Furthermore, every individual in Chinese society wants sustainability in the development and implications of environmental strategies. In this regard, the more reliable organizations would work more effectively in providing a more reliable higher physical education to the community and the employees considering CSR’s collective goals. Therefore, the practical implications of this study are important to consider because, with the help of these implications, it would be more suitable and understandable for the stakeholders to work for environmental sustainability, which is a major problem not only for China but also for every corner of the world where the environmental issues prevail. In other words, the implications of this study would not be limited to the context of China. However, the study’s findings also apply to every country and organization worldwide.

This research has significant effects on legislators, educators, environmentalists, and technology developers. First, this study may help Chinese officials create and execute environmental sustainability policies. Policymakers should invest in educational programs that incorporate sustainability and technology by acknowledging the favorable link between modern physical education growth, innovation in technology, and the preservation of the environment. They may also encourage the adoption of eco-friendly technology across industries to assist sustainable growth. Educators shape future generations’ views and behaviors. Thus, this investigation emphasizes the need to include environmental and technology literacy in school curricula. By introducing sustainability-focused modules into physical education programs and teaching students how technology breakthroughs may help the environment, educators can empower students to transform their communities.

This research may help environmentalists promote sustainable practices and technology in numerous areas. Advocates may urge firms and industries to prioritize eco-friendly practices and invest in sustainable technology research by emphasizing the beneficial association between technological innovation and environmental sustainability. They may work with schools to enhance environmental awareness and encourage environmentally responsible behavior among students and the community. Technology developers are essential to sustainable innovation. This research shows that technology developers must prioritize solutions that meet social demands and promote environmental sustainability. By investing in green power systems and eco-friendly materials, technology developers may reduce environmental issues and create a more sustainable future. The findings may help stakeholders promote sustainability and innovation for future generations.

For educational technologists and physical education educators, this research has significant outcomes. Educational technologists may use this knowledge to create novel educational interventions that combine advanced physical education with technology innovation to improve environmental sustainability. Interactive digital platforms, virtual simulations, and immersive learning experiences may help educational technologists engage students and teach about environmental challenges and sustainable practices in physical education. Digital tools and apps may also give personalized feedback and track physical activity levels, encouraging pupils to be healthy and environmentally conscientious. This research may also help physical education trainers improve their teaching and curriculum. Trainers may encourage better lives and environmental responsibility by emphasizing the links between physical exercise, technology innovation, and sustainability. Trainers may also use wearable activity trackers, augmented reality, and gamification to improve physical education programs. This research shows that educational technology and creative pedagogical methods may revolutionize physical education to achieve environmental sustainability objectives.

## Conclusions

This study was designed to understand the role of higher physical education development and technological innovation in attaining a sustainable environment in China. In this regard, this study has significant implications that are important to be considered for improving the sustainability of the environment in China. Also, it is critical to understand that the sustainability of the environment is not easy to achieve. However, it is the responsibility of all the stakeholders of the environment, including Organizations and the government, to develop strategies effectively for a better understanding of the environmental problems and provide meaningful solutions to address the environmental problems in China. Given that, the current study was designed to provide a detailed insight into the relationship of different variables that are taken to address the theoretical gap in the literature and the practical gap in implications, as without the fulfillment of these gaps, it would be a lame idea for the organizations to proceed further on the road towards the sustainability of the environment. In this way, the study concludes that higher physical education has a crucial role in developing and advancing environmental sustainability. It is also observed that higher physical education helps in a better understanding and appropriate mental development, which are critical for a successful and sustainable mental development that triggers people to think critically about the sustainable development of the environment. Furthermore, this study concludes that compatibility and simplicity are important in the sustainable development of the environment, yet earlier studies did not address these factors. Similarly, this study demonstrates the important mediating role of technological innovation sustainability in developing environmental sustainability in the context of China, which may apply to other countries that are similar in context to China.

This research affects physical education sustainability and technology innovation scholars as well as practitioners. The study illuminates the complex linkages between advanced physical education development, technology innovation, and environmental sustainability, laying the groundwork for future research. This study may help sustainability researchers understand how educational interventions and technological advances promote sustainable behaviors and attitudes. Integrating sustainability ideas into the physical education curriculum might also inspire multidisciplinary environmental research partnerships. Technological innovation academics may also examine how Artificial Intelligence (AI), Virtual Reality (VR), and the Internet of Things (IoT) might improve educational sustainability projects. Researchers may use sustainability and technology innovation to create creative physical education solutions that promote environmental awareness and stewardship by creating cross-disciplinary communication and cooperation. This study offers valuable insights and options for future research on sustainability and technology for good social change.

### Limitations and future directions

This research examines how enhanced physical education and technological innovation promote ecological sustainability in China. However, to better comprehend this complicated interaction, we must admit limitations and suggest future research areas. This research discounts additional factors that may moderate or mediate the association between physical education, breakthroughs in technology, and preserving the environment. Future studies might examine how social conventions, financial regulations, and organizational structures moderate environmental sustainability educational and technological initiatives. Investigating how technology innovation mediates the link between physical education and a healthy environment may reveal the processes behind behavioral change and sustainable conduct. This research emphasizes the significance of technical innovation in environmental sustainability. However, it is essential to recognize the risks and downsides of fast technological growth. Technological innovation’s unforeseen effects on energy consumption, digital pollution, and electronic waste should be studied for environmental sustainability.

Furthermore, studying the equity implications of technology innovation in education and environmental sustainability resources might help eliminate inequities and promote inclusive and equitable results for everyone. The study also concentrates on China, restricting its applicability to other nations with varied socioeconomic, financial, and ecological circumstances. Future studies might compare how physical education, innovative technology, and environmental conservation vary between regions. By analyzing cross-national variances and similarities, investigators may find global environmental sustainability best practices and policy suggestions.

## Data Availability

The data supporting the findings of this study corresponding author (lsp20201214@zzu.edu.cn) upon reasonable request.
